# Description and design considerations of a randomized clinical trial investigating the effect of a multidisciplinary cognitive-behavioural intervention for patients undergoing lumbar spinal fusion surgery

**DOI:** 10.1186/1471-2474-15-62

**Published:** 2014-03-03

**Authors:** Nanna Rolving, Lisa Gregersen Oestergaard, Morten Vejs Willert, Finn Bjarke Christensen, Frank Blumensaat, Cody Bünger, Claus Vinther Nielsen

**Affiliations:** 1 Department of Physical and Occupational Therapy, Aarhus University Hospital, Aarhus, Denmark; 2 Department of Occupational Medicine, Aarhus University Hospital, Aarhus, Denmark; 3 Institute of Clinical Medicine, Aarhus University Hospital, Aarhus, Denmark; 4 Public Health and Quality Improvement, Aarhus, Central Denmark Region, Denmark; 5 Department of Orthopedics, Aarhus University Hospital, Aarhus, Denmark

**Keywords:** Spine surgery, Chronic low back pain, Rehabilitation, Cognitive-behavioural therapy, Randomized clinical trial

## Abstract

**Background:**

The ideal rehabilitation strategy following lumbar spinal fusion surgery has not yet been established. This paper is a study protocol, describing the rationale behind and the details of a cognitive-behavioural rehabilitation intervention for lumbar spinal fusion patients based on the best available evidence. Predictors of poor outcome following spine surgery have been identified to provide targets for the intervention, and the components of the intervention were structured in accordance with the cognitive-behavioural model. The study aims to compare the clinical and economical effectiveness of a cognitive-behavioural rehabilitation strategy to that of usual care for patients undergoing lumbar spinal fusion surgery.

**Methods/Design:**

The study is a randomized clinical trial including 96 patients scheduled for lumbar spinal fusion surgery due to degenerative disease or spondylolisthesis. Patients were recruited in the period October 2011 to July 2013, and the follow-up period is one year from date of surgery. Patients are allocated on a 1:2 ratio (control: intervention) to either treatment as usual (control group), which implies surgery and the standard postoperative rehabilitation, or in addition to this, a patient education focusing on pain behaviour and pain coping (intervention group). It takes place in a hospital setting, and consists of six group-based sessions, managed by a multidisciplinary team of health professionals.

The primary outcomes are disability (Oswestry Disability Index) and sick leave, while secondary outcomes include coping (Coping Strategies Questionnaire), fear-avoidance belief (Fear Avoidance Belief Questionnaire), pain (Low Back Pain Rating Scale, pain index), mobility during hospitalization (Cumulated Ambulation Score), generic health-related quality of life (EQ-5D) and resource use. Outcomes are measured using self report questionnaires, medical records and national registers.

**Discussion:**

It is expected that the intervention can provide better functional outcome, less pain and earlier return to work after lumbar spinal fusion surgery. By combining knowledge and evidence from different knowledge areas, the project aims to provide new knowledge that can create greater consistency in patient treatment. We expect that the results can make a significant contribution to development of guidelines for good rehabilitation of patients undergoing lumbar spinal fusion.

**Trial registration:**

Current Controlled Trials ISRCTN42281022.

## Background

For sufferers of chronic and severe back pain, lumbar spinal fusion (LSF) surgery is a commonly offered treatment strategy when conservative treatment has failed [1,2]. In the past two decades reports have indicated significant increases in spinal fusion rates in the western countries, with the largest increase seen in the US. Here the annual age and sex adjusted rates of LSF procedures increased with 220% in the period 1990 – 2000 [3] and another 170% in the period 1998–2008 [4]. These rises are in part due to advances in surgical fusion techniques and devices, e.g. the approval of intervertebral fusion cages in 1996, intended to improve postoperative outcomes [5,6]. The increasing number of LSF operations and the consequent increased costs and risk of complications and adverse events [6-9] may be justified by the benefits of the procedure. But the efficacy of LSF for degenerative spinal disorders has not yet been established, as published reviews on the subject disagree [10-12]. One review including randomized, nonrandomized and retrospective studies concluded that the body of literature supports LSF as a viable treatment option for chronic LBP [11]. Two other reviews included only randomized controlled studies (RCT) comparing LSF to conservative treatment [10,12]. The earliest review concluded that robust conclusions on the efficacy of LSF could not be made. In the latter publication the authors stated that there is strong evidence that LSF is no more effective than conservative treatment. In both reviews the comparable effect of the conservative treatment was in evidence only with the use of structured rehabilitation with a cognitive-behavioural approach (CBT). Whether an additive effect can be achieved by combining LSF with a postoperative rehabilitation strategy applying CBT has been investigated in only one study [13]. Here, LSF followed by a 12 week intervention combining CBT and a structured exercise program resulted in significantly better outcomes on disability, pain and return to work compared to LSF plus standard rehabilitation. The standard rehabilitation consisted of a 12 week home based exercise program, commencing with one instruction from the physical therapist at the hospital at discharge. Another RCT including behavioural elements in the rehabilitation intervention following LSF similarly found benefits of the intervention, compared to both 8 weeks supervised exercise and to a video-guided home-based exercise program similar to that of Abbott et al. [14].

The idea of using CBT in combination with LSF seems rational, as psychosocial factors have become increasingly accepted as potentially important determinants of outcomes following spine surgery [15-18]. In particular, characteristics such as maladaptive coping strategies, fear-avoidance beliefs, preoperative anxiety and pain catastrophizing seem to be predictive of worse outcomes in pain, function and quality of life after surgery [15-18]. Modifying these traits, and thereby improving pain levels, disability and quality of life, seems to be achievable through the application of CBT [13,19-21].

An additional two parameters seem important in successful rehabilitation of surgically treated CLBP patients. The first is the management of the (CBT) rehabilitation by a multidisciplinary team, which two studies have found to be an important key factor in a population of severely disabled CLBP patients [22,23]. However, no studies including both CBT and multidisciplinary management in the rehabilitation strategy were found. The second parameter concerns the timing of rehabilitation. In the published studies on LSF patients, all but one initiate the rehabilitation intervention after surgery. It may be questioned, though, whether a better effect can be achieved by initiating an intervention already prior to surgery. One study by Nielsen et al. [24] investigated the effect of prehabilitation and early postoperative rehabilitation (presurgical training, analgesics and nutrition) compared to standard care. Patients in the intervention group reached the recovery milestones faster and left hospital earlier. A number of studies on preoperative interventions for populations of hip- and knee arthroplasty patients point to the same result, namely a benefit of initiating the intervention already prior to surgery [25,26]. None of these studies involve CBT or multidisciplinary strategies in their (p)rehabilitation.

On this basis we find it relevant to design a multidisciplinary rehabilitation strategy using a CBT approach, initiated prior to fusion surgery. The purpose of this paper is to describe the theoretical basis and the details of the intervention used in the study. This is in accordance with international recommendations for the development and evaluation of complex interventions in clinical trials [27].

### Defining cognitive-behavioural therapy

The biopsychosocial approach of CBT focuses on the complex interplay of cognitive, emotional, behavioural and social factors and how they interact with the biomedical factors. The main assumption is that a person’s thoughts and beliefs about their problem will influence their feelings and physiologic reactions and their consequent behaviours [28-30]. The role of behaviour is important, as people often act in ways that serve to maintain the unhelpful beliefs of the individual, and in this way a vicious circle persists. This strong link between beliefs predicting behaviour has been shown in several studies in CLBP patients [31,32]. Consequently, the goal of cognitive behavioural therapy is to identify and challenge maladaptive thoughts, and consequently modify feelings and behaviours, and thereby the experience of pain. The basic assumptions about pain behaviour and the relevant techniques applied for cognitive and behavioural therapy are as follows.

*The cognitive aspect* of CBT is based on Beck’s cognitive model promoting the idea, that a person’s cognition has an impact on their mood and emotions, their bodily reactions and their behaviour [28]. On a general level, three cognitive levels can be described. They are 1) (negative) automatic thoughts, being thoughts that surface quickly and automatically when a person is in a particular situation, e.g. “my boss will think less of me”, when being late for a meeting 2) Underlying assumptions, being unconscious and unspoken assumptions that govern our everyday behaviour. They can be positive, e.g. “if I achieve well in school, my parents will respect and love me” and correspondingly negative “If I fail this test I will make my parents unhappy and they will dislike me”. These assumptions cause us to live by rules, often expressed as what one “ought to” or “should” do to fulfill the underlying assumptions. 3) Schematas, being our fundamental perception of our selves, other people and the context in which we exist. A person usually possesses both positive (I’m respectable/lovable/trustworthy) and negative (I’m boring/unintelligent) schematas. The techniques used in cognitive therapy focus mainly on intervening on the first level, namely the thoughts, beliefs and expectations, that contribute to the negative emotions associated with chronic pain. Therefore the major goals of cognitive techniques are 1) help the patient become aware of how negative thoughts affect their mood, behaviour and pain and 2) challenge and modify the thoughts and through that promote improved pain coping. The techniques frequently applied are cognitive restructuring, problem solving, distraction and prevention of relapses.

Where the focus of the cognitive elements are on thoughts, beliefs and expectations, the main focus of *the behavioural aspect* is on a person's behaviours. The notion of behavioural therapy lends from the learning principles of operant conditioning, maintaining that social learning processes may account for instances where pain behaviours persist when healing could have occurred [33]. For instance, a worried parent may reinforce a pain behaviour of activity avoidance in his or her child. The two major goals of the behavioural therapy are hence 1) to increase the frequency of adaptive, well-suited behaviours and 2) to decrease maladaptive pain behaviours. Behavioural techniques used to achieve these goals are activity pacing, scheduling of pleasant activities, time-contingent medication and social reinforcement.

#### ***Catastrophizing and fear-avoidance belief***

**Figure 1:**
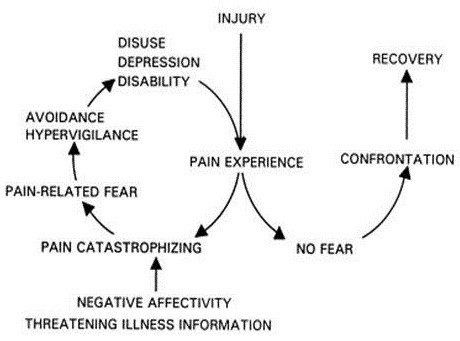
**The fear-avoidance belief model (Vlayen & Linton **[35]**).**

As can be summarized from the above description, a person’s negative beliefs, thoughts and expectations may cause them to behave and cope in a maladaptive manner in relation to their pain. Particularly fear-avoidance belief, catastrophic thinking and a feeling of helplessness and lack of control seem to be associated with passive coping strategies like rest and avoidance behaviour. The fear-avoidance model perceives catastrophic thinking as a prerequisite and an elementary factor for the development of avoidance behaviours [34]. It suggests how individuals with negative beliefs about pain will have a pain perception that is imbued with catastrophic interpretations. In an attempt to avoid this perceived catastrophic threat the person engages in avoidance behaviour, becoming gradually more deconditioned and disabled. As the patient engages in avoidance behaviour, he avoids an increasingly larger array of movements and activities and may spend a lot of time resting. The use of such passive (maladaptive) coping strategies may delay or in the worst case obstruct rehabilitation of the patient after the operation. The parts of the back affected by the operation (ligaments, muscles, nerves) will not gain the necessary strength and flexibility, if not exposed to a gradually larger strain according to recommendations. The tissue becomes weak and more prone to overloading, which again causes pain. Over time the patient may become increasingly more disabled and limited in work and social life with a consequent reduction in their quality of life. The fear-avoidance model, as presented by Vlayen and Linton [35] is depicted in Figure 1. As described earlier, interventions that have used CBT approaches to target catastrophizing and fear-avoidance behaviours have shown an association with a reduction in pain behaviours, physical disability and depression in both surgical and non-surgical CLBP patients [13,20,21,36,37].

#### ***Targets for a multidisciplinary CBT intervention for patients undergoing LSF surgery***

Summarizing the theoretical background and the research described above, the targets of the intervention should encompass the following basic components.

• The first component aims to increase the patient’s knowledge about pain and pain perception in order to gain a better understanding of how cognitions and behaviour affect the pain experience. It emphasizes the role that the patient can play in controlling his or her own pain.

• The second component relates to the use of active coping skills. Skills training may use a variety of cognitive and behavioural pain-coping strategies, such as activity pacing and pleasant activity scheduling. Catastrophic pain-related thoughts are challenged by formulating more realistic appraisals using cognitive restructuring techniques.

• The third component involves the application and maintenance of learned coping skills. The patients are now encouraged to try out the learned coping skills to an increasingly wider range of daily situations.

• The fourth component focuses on problem-solving methods that enable the patients to analyse and develop plans for dealing with pain flares and other challenging situations.

## Methods/Design

### Trial design

The study is a randomized clinical trial with 1-year follow-up.

### Participants

Patients eligible for inclusion were persons at the age of 18–64 years scheduled for lumbar spinal fusion surgery (maximum of 3 adjacent vertebrae) due to pain and disability as a consequence of degenerative diseases or spondylolisthesis grade 1 or 2. Patients were included from two hospitals in the same uptake area covering 1,272,510 inhabitants.

### Interventions

#### ***Control group***

Patients in the control group received usual care, being the department's standard care related to their surgical procedure. In one of the hospitals usual care included a voluntary information meeting for patients and their spouses/relatives, approximately 2 weeks prior to surgery. Patients were routinely called for a control visit with the operating surgeon at 3 and 12 months. Following the 3 months control visit, patients commenced physical rehabilitation in their local community (e.g. physical therapy clinic or rehabilitation centre). This typically consists of 1–2 individual sessions followed by 10 sessions of supervised group-based exercise.

#### ***Intervention group***

In addition to the described usual care (surgery and physical rehabilitation), patients in the intervention group participated in a patient education using a cognitive-behavioural therapy (CBT) approach.

##### 

**Structure** The CBT intervention consisted of six sessions, each of three hours duration. Patients were to attend four of the sessions prior to surgery, while the fifth and sixth sessions were placed postoperatively, at three and six months respectively. The length of the intervention was designed to optimize attendance with the time required to develop the needed skills. A previous study found no difference in outcome between programs of 15, 30 or 60 hours duration [38]. A group format was used for the intervention, as evidence suggests that there is no difference in outcome between individual and group therapy [39].

##### 

**Setting** The six sessions of the patient education took place in a conference room at the hospital, where most of the patients were to undergo surgery. The room was placed in an administrative building and hence did not have the look of a typical hospital ward. The room was big enough for the participants to move around, sit or stand as they pleased.

##### 

**The multidisciplinary team** The health professionals delivering the intervention were a psychologist, an occupational therapist, a physiotherapist, a spine surgeon, a social worker and an experienced patient. The psychologist and occupational therapist both attended the sessions most of the time, and one or the other was always present at the attendance of the other health professionals. The focus of their roles concerned the presentation of a unified message rather than the maintenance of traditional professional boundaries. The surgeon and the two therapists were affiliated to the surgical department at the hospital where the intervention took place, and all had several years of experience with LSF patients. The psychologist and social worker both had several years of experience with multidisciplinary treatment of chronic back pain patients in a hospital setting. The team participated in a 2-day training program, covering the risk factors associated with chronic pain, the CBT model, developing basic CBT skills including questioning techniques, and learning the topics to be covered in each session. Training was delivered by the psychologist participating in the intervention. Furthermore an intervention manual forming the intervention guidelines was developed in order to standardize the intervention as much as possible.

##### 

**Content** The content of each session was pre-specified with some flexibility to respond to participants’ needs. Details of each session are presented in Table 1. The key elements of the contents of each session were summarized in a patient handbook handed out the patient’s first attendance. The post-operative sessions focused on recapitulation of the CBT tools learned at the preoperative sessions, and how they could be used in the rehabilitation process after the operation. There was left sufficient time for patients to discuss worries and unexpected up- and downturns after the operation, and to share their individual experiences regarding the learned pain coping strategies with fellow patients. The slide show used in the group sessions, the patient handbook and the intervention manual can be retrieved by contacting the first author of this paper.

**Table 1:** Contents of each of the six sessions

***All sessions commence with a short presentation of the day's session, new participants are introduced, and homework and questions from the previous session are discussed.***	
	**Session A – preoperative**
**CBT**	Physical and psychological reactions in stressful situations.
	The link between thoughts, feelings, bodily reactions and behaviour.
**Preparing for surgery**	What to expect of the operation and the postoperative course.
**Homework**	Identify and write down thoughts and feelings in relation to painful or stressful situations. Consider and write down alternative and realistic thoughts.
	**Session B – preoperative**
**CBT**	Causes and consequences of pain. The fear-avoidance belief model and the importance of physical activity in reducing pain.
**Preparing for surgery**	Pleasant activity scheduling and activity pacing.
	Ergonomics - working posture following surgery.
**Homework**	Identify and write down 3 activities you used to enjoy. Plan and go through with them considering your pain level. How did it affect your mood and pain level?
	**Session C – preoperative**
**CBT**	The link between thoughts, feelings, bodily reactions and behaviour.
	Negative automatic thoughts and their role in maintenance of a vicious circle.
	Active and passive coping strategies.
**Homework**	Identify and write down your own coping strategies when in pain and distress.
	Try to use active coping strategies. How did it affect your pain level?
	**Session D – preoperative**
**CBT**	How to cope with pain and distress in relation to family, friends and work.
**Preparing for surgery**	The experiences of a previously operated patient.
	Legislation and procedures in the authorities when being on sick leave and in relation to return to work.
**Homework**	Say no to 3 tasks, that you would usually agree to do, despite not being comfortable doing it.
	Promt a friend, colleague or family to give you a positive support remark.
	Give a friend, colleague or family a positive remark and notice the reaction.
	**Follow-up session 1 – postoperative (3 months)**
**CBT**	Reflection of how patients have used the acquired cognitive techniques and coping strategies postoperatively.
	Using pacing techniques to restart daily activities, hobbies and work.
**Homework**	Goal setting for the next three months.
	Use pacing techniques to achieve one or more of your goals.
	**Follow-up session 2 – postoperative (6 months)**
**CBT**	Reflection of how patients have used the acquired cognitive techniques and coping strategies during the past 3 months.
	Discussion of achievements of previously set goals. Setting future goals.
	Coping with flare-ups.
	Returning to work – expectations, worries and how to cope with barriers.

### Outcomes

For each patient baseline characteristics will be registered by questionnaires or medical records: gender, age, working status, diagnosis, type of operation, comorbidity, and previous spine surgery.

#### ***Primary outcomes***

– Disability (Oswestery Disability Index) [40].

– Sick-leave/Return to work.

#### ***Secondary outcomes***

– Pain (Low Back Pain Rating Scale) [41].

– Quality of life (EQ-5D) [42].

– Coping strategies (Coping strategies questionnaire) [43].

– Fear-avoidance (Fear-avoidance beliefs. questionnaire) [44].

– Readmission to hospital and use of health care services.

Data concerning the patients’ use of health care service and return to work is based on self-registration [45]. Data on sick-leave compensation will be obtained from the DREAM Database, a national database administered by the Ministry of Employment.

### Sample size and data analysis

Based on a pilot study, a standard deviation of the ODI was set at 14 points on the ODI scale. A 10 points difference has been estimated as clinically relevant in a study comparing LSF with cognitive intervention [46]. Significance level was set at 0.05 and the power at 0.80. To fulfill these criteria, the study would need at total of 72 patients. With the 2:1 randomisation ratio this would mean 48 patients in the intervention group and 24 in the control group. If assuming that 10% of the included patients will be lost to follow-up, at least 80 patients should be included in the study.

All data are entered in EpiData twice. STATA 12.0 will be used for statistical evaluation. Parametric tests will be used for normally distributed data and non-parametric tests for data which are not normally distributed. Because the ODI and EQ-5D are ranked data, the Wilcoxon rank sum test will be used to test any differences between the groups within these questionnaires. The differences between numerical data (length of stay at hospital, use of health care service, physical function) will be tested with Two Sample *t*-test or Wilcoxon rank sum test. The intervention group is tested against the control group.

### Cost effectiveness and cost utility evaluation

The economic evaluation alongside the study will be conducted as a cost-effectiveness analysis with the main parameter being ODI and incremental costs per earlier day of RTW. The EQ-5D data valued by the Danish set of preference weights will qualify as a cost-utility analysis reporting the cost per quality-adjusted life-year (QALY) [36]. The perspective of the analysis will include the primary and secondary health care sector, the patient perspective, and the societal perspective. Resource utilisation will be informed by ad hoc data collected using a modified version of the Dutch cost diary [45]. Valuation will be based on micro-costing (intervention costs), Diagnosis-Related-Grouping (DRG) tariffs (other contacts in secondary health care), the collective agreement between Local Government Denmark and primary care practitioners (contacts in primary care), market prices (patients’ costs) and national average gross salaries (production loss and time costs in general). Statistical tests will be based on precision estimates (confidence intervals) calculated using the technique of bootstrapping [47].

### Ethical considerations

The intervention carries no risk to the patients who as a minimum are ensured the standard treatment. The study will be carried out in accordance with Declaration of Helsinki and Good Clinical Practice. The study has been approved by the Danish Protection Agency and The Ethical Committee of Central Denmark Region (journal no. M-20110047).

## Discussion

In the future an increasing proportion of older people have to live with chronic diseases. Knowledge of new interventions is required to achieve greater individual and societal gains. This project combines knowledge and evidence from different knowledge areas (biomedicine, psychology, physiotherapy and occupational therapy) because the areas isolated do not have optimal effect and because of increasing evidence of efficacy of integration of more of the areas. Further we include health economic analyses, which we hope further can provide prioritization in health and social planning. We hope that our intervention can provide better functional outcome, less pain and earlier return to work after surgery. The project is expected to provide new knowledge that can create greater consistency in patient treatment. We expect that the results can make a significant contribution to development of guidelines for good rehabilitation of patients undergoing lumbal spinal fusion. Further that new knowledge can inspire similar developments within a wide range of diseases.

## Abbreviations

LSF: Lumbar spinal fusion; CBT: Cognitive-behavioural therapy; ODI: Oswestry disability index; EQ-5D: EuroQol 5 dimensions.

## Competing interests

The authors declare that they have no competing interests.

## Authors’ contributions

NR participated in the design planning and coordination of the study. NR furthermore participated in the development and planning of the CBT intervention. NR drafted the manuscript. LGO conceived of the study and participated in its design. LGO also helped develop the CBT intervention and revised the manuscript. FB and MVW participated in the development of the CBT intervention and revised the manuscript. CVN has participated in the design of the study and has revised the manuscript. FBC and CB have revised the manuscript. All authors have read and approved the final manuscript.

## Pre-publication history

The pre-publication history for this paper can be accessed here:

http://www.biomedcentral.com/1471-2474/15/62/prepub
